# “Commentary: Alcohol Consumption Impairs the Ependymal Cilia Motility in the Brain Ventricles”

**DOI:** 10.29245/2572.942X/2019/2.1250

**Published:** 2019-04-14

**Authors:** Hannah C. Saternos, Wissam A. AbouAlaiwi

**Affiliations:** 1 University of Toledo, College of Pharmacy and Pharmaceutical Sciences, Department of Pharmacology and Experimental Therapeutics, Toledo, Ohio, USA

In a recent article, Al Omran et al. investigate the mechanism behind cerebral spinal fluid (CSF) circulation in the brain^1^. The movement of CSF around the nervous system is not well understood but is generally believed to circulate due to forces generated by the cardiopulmonary system. The authors present a unique perspective and novel mechanism suggesting motile cilia motion is responsible for the movement of CSF and that ependymal cilia dysfunction may contribute to abnormal CSF circulation observed in brain injury, specifically hydrocephalus. To assess ependymal ciliary motion in the brain ventricles, the group developed a new imaging technique.

Often associated with excess alcohol consumption, hydrocephalus is characterized by an increase in CSF production and decreased absorption leading to a build-up of fluid in brain cavities such as the ventricles. The excess fluid causes the cavities to swell putting pressure on key regions of the brain leading to symptoms observed in these patients. The movement of CSF from the choroid plexus throughout the nervous system is fundamental for its absorption^2^, Al Omran suggests that motile cilia not only are responsible for CSF circulation, but that ethanol hinders the movement of ependymal motile cilia, preventing proper fluid flow and thus CSF absorption into the blood stream. Cilia involvement in hydrocephalus has been an openly debated topic largely bolstered by prior imaging methods that made it difficult to directly visualize the dynamics of motile cilia in tissues. Al Omran et al. developed a new live imaging technique to observe motile ependymal cilia and were the first to capture their unique movement patterns in the brain ventricle *ex vivo*. The success of this work is mostly attributed to this new method, which enables researchers to directly observe and measure parameters involved in motile cilia motion in a variety of tissues such as the brain, reproductive organs, and lung airways. However, this method is not without its shortcomings. The most notable being the choice between tissue viability time and tissue thickness. Under the right conditions, the tissue life can be extended upwards of 2 hours. For maximal time, hand slicing the tissue is the preferred method in this article but is difficult and heavily relies on personnel skill level. Hand slicing leads to thicker tissue samples reducing image quality under the microscope but does allow quick access to the ventricles. This method keeps the tissue in solution longer thus maintaining viability for longer. It is possible to section the brain with a vibratome which will provide thinner slices leading to clearer images but requires the tissue to be out of solution for longer, thus reducing the time before the tissue degrades. There is also a decision about accuracy, compared to a vibratome, which allows for more accurate region identification, hand slicing requires the researcher to blindly cut into the tissue risking damage to the ventricle or incorrect sectioning. However, these alternative options allow for customization of the technique to best suit the experimental design enabling a broader application of this technique to both basic and clinical sciences. From a basic science perspective, improved visualization techniques will lead to an enhanced understanding of the functional role of motile cilia not only in the movement of cerebrospinal fluid but also to applications in other areas of motile cilia research. The clinical implications associated with new insights into motile cilia dynamics in the body could translate into novel therapeutic targets and new research strategies for diseases related to cilia motion.

By using this method, Al Omran et al. discovered three distinct movement patterns of motile cilia within the brain ventricle providing evidence on how these organelles function and circulate fluid throughout the body. The authors interpreted the variations in beating frequency and angle to be indicative of the existence of three distinct subtypes of motile cilia that specifically localize to different regions within the brain ventricle. Although the variations can be clearly observed and measured, these differences could be due to the respective cellular localization and angle within the brain ventricle. The study lacks investigation into potential structural or motor differences between the three ciliary sub-types, a limitation the authors acknowledge but makes definitive conclusions about motile cilia classification premature. Tissue viability, using hand slicing in the live imaging technique, can be extended towards two hours under the right conditions; however, tissue viability begins to decline immediately after death. The authors claim a reduction in cilia beating frequency after ethanol exposure, but it is not clear whether accurate controls or proper comparisons were made to draw this conclusion. The study design directly compared beating frequency before and after ethanol exposure meaning ethanol was introduced to the tissue an hour after dissection; thus, tissue viability and decline could be a significant contributing factor for the reduction in beating pattern over time, not ethanol exposure. Proper controls to monitor the changes in cilia motion as the experimental time approaches two hours is crucial to fully support or disprove the involvement of alcohol on disrupted cilia motion at least in the *ex vivo* experiments.

In conclusion, the technique developed is a drastic improvement on prior imaging methods having implications to improve the field of motile cilia research in general. The evidence presented is suggestive of three distinct motile cilia classes but a more thorough characterization of structural differences as well as appropriate controls are needed before any definitive conclusions can be made about classification or role in CSF circulation. The evidence presented does suggest a potential connection and further investigation into the role of motile cilia and hydrocephalus is needed and the live imaging technique described here affords researchers with an additional tool to investigate the role of motile cilia.
